# Sex-Dependent and Asymmetric Associations of Bodyweight History in the Twenties with Later HbA1c Trajectories in a Japanese Occupational Cohort

**DOI:** 10.3390/nu18101532

**Published:** 2026-05-12

**Authors:** Katsumi Iizuka, Eri Hiraiwa, Hitomi Matsuura, Kotone Yanagi, Kiyomi Kaito, Kanako Deguchi, Hiroyuki Naruse

**Affiliations:** 1 Department of Clinical Nutrition, School of Medicine, Fujita Health University, Toyoake 470-1192, Japan; 51022099@fujita-hu.ac.jp (E.H.); 51023103@fujita-hu.ac.jp (H.M.); kanasakuran@gmail.com (K.D.); 2 School of Medicine, Fujita Health University, Toyoake 470-1192, Japan; 3 Health Management Center, Fujita Health University, Toyoake 470-1192, Japan; yanagi-k@fujita-hu.ac.jp (K.Y.); kkaito@fujita-hu.ac.jp (K.K.); hnaruse@fujita-hu.ac.jp (H.N.)

**Keywords:** underweight, overweight, HbA1c, glycemic trajectory, longitudinal study, occupational cohort

## Abstract

**Background:** Underweight status is common among young women in Japan and has been linked to impaired glucose tolerance, but its long-term association with HbA1c trajectories remains unclear. This study examined whether body size history in the twenties is associated with subsequent HbA1c trajectories across adulthood. **Methods:** We analyzed health check-up data from Fujita Health University, collected between 2003 and 2025. Participants were classified as normal weight in the twenties (NW20s), underweight at least once in the twenties (UW20s_ever), or overweight at least once in the twenties (OW20s_ever), excluding mixed underweight/overweight histories. Eligible individuals had at least 5 years of follow-up, at least five BMI and HbA1c measurements, and at least one BMI record between ages 20 and 29 years. HbA1c trajectories were evaluated using sex-stratified linear mixed-effects models. Kaplan–Meier and Cox regression analyses were used to assess the risk of first reaching HbA1c ≥ 5.6%. **Results:** A total of 2923 participants were included in the trajectory analysis. For the time-to-event analysis, 2753 participants were included after exclusion of 170 participants with HbA1c ≥ 5.6% at study entry. Body size history in the twenties was associated with distinct, sex-specific HbA1c trajectories. OW20s_ever showed persistently higher HbA1c levels in both women and men, but the local slope of HbA1c was greater at ages 35 and 45 years in women and at age 25 years in men. In contrast, UW20s_ever showed lower HbA1c levels than NW20s at ages 25 and 35 years only in women. In complementary time-to-event analyses, OW20s_ever was associated with a higher risk of HbA1c ≥ 5.6% in both women and men (women: HR 1.37, 95% CI 1.06–1.76, *p* = 0.016; men: HR 1.81, 95% CI 1.41–2.32, *p* < 0.001), whereas UW20s_ever was associated with a lower risk only in women (HR 0.81, 95% CI 0.67–0.98, *p* = 0.028). **Conclusions:** Underweight and overweight history in the twenties are not simply mirror-image exposures but rather have sex-dependent and asymmetric associations with later HbA1c regulation.

## 1. Introduction

In Japan, the prevalence of being underweight among young women is high, thus demonstrating this factor as an important issue both clinically and from a public health perspective [1,2,3]. An underweight status among young women is known to be associated with infertility, low birth weight, decreased bone mass, and increased risks of osteoporosis [1,2,3,4,5,6,7,8,9,10,11,12,13,14,15,16]. Underweight women are more likely to give birth to low-birth-weight babies, and these children are more prone to developing diabetes and high blood pressure [4,15,16].

Furthermore, we reported that an underweight status among young women may be accompanied by imbalances or deficiencies in nutrient intake, reduced muscle strength, and decreased lymphocyte count [8,9]. We have also observed that the gut microbiota of these individuals differs from that of individuals with normal weights [10]. In recent years, concepts such as female underweight/undernutrition syndrome (FUS) have been proposed by some academic societies to increase the social awareness of underweight status among young women [17]. On the other hand, our recent results have also shown that among people in their twenties who were told even once that they were underweight, only 40% remained underweight in their thirties and beyond [18].

Conversely, the association between body constitution and the risk of glucose intolerance or diabetes is well established, and it is widely known that obesity (especially visceral fat accumulation) is a major cause of impaired glucose metabolism [19,20,21,22,23,24]. Furthermore, in Asian individuals, the compensatory increase in insulin secretion in response to weight gain may be relatively small. Therefore, even in nonobese individuals, if visceral fat accumulation is present, glucose tolerance may deteriorate because of insulin resistance [10,21,24,25]. Numerous longitudinal studies have also reported that weight gain is associated with increases in HbA1c levels and the incidence of glucose intolerance [26,27,28].

However, interpretations of the relationship between “underweight” in young women and the risk of glucose metabolism disorders may vary depending on the evaluation index used. For example, a report focusing on underweight young Japanese women revealed that the frequency of impaired glucose tolerance (IGT), assessed using a 75 g oral glucose tolerance test (OGTT), was higher than that in women of normal weight (13.3% vs. 1.8%) [29]. In contrast, in some cross-sectional data, when HbA1c was used as the indicator, underweight young women did not exhibit a clear difference in HbA1c levels compared with women of normal weight [30,31]. Because the OGTT is conducted with a fixed glucose load (75 g for adults), the relative load may be greater in a group with a smaller body size. Moreover, as the OGTT assesses short-term glucose tolerance (while HbA1c reflects average blood glucose over a longer period of time), discrepancies may arise between OGTT-based IGT findings and those based on HbA1c.

Furthermore, many of these findings are based on cross-sectional studies, and investigations into how differences in body type affect changes in HbA1c over time (trajectory) within individuals have not been sufficient. In addition, for women, whether fat distribution and glucose tolerance change through alterations in the hormonal environment after the menopausal transition is a topic of debate [32,33,34], thus indicating the need for longitudinal evaluations [35,36].

On the basis of the abovementioned background, this study classified subjects into three groups according to their BMI history in their twenties: those who maintained normal weight throughout their twenties (NW20s), those who were underweight at least once in their twenties (UW20s_ever), and those who were overweight at least once in their twenties (OW20s_ever). Our recent findings further suggested that underweight status in young adulthood is often concentrated around the lower boundary of normal weight and may fluctuate across examination years. Therefore, a history-based classification may better capture the relevant phenotype of young-adult underweight than a one-time cross-sectional definition [18]. After stratification by sex, we evaluated longitudinal HbA1c trajectories using estimated marginal means (EMMs) and local slopes, and we also examined the time to first occurrence of HbA1c ≥ 5.6% using the Kaplan–Meier method and Cox regression analysis within the same study framework. By combining trajectory-based and threshold-based analyses, we aimed to clarify whether bodyweight history in the twenties shows sex-dependent and potentially asymmetric associations with later HbA1c regulation. Clarifying these relationships may help refine strategies for maintaining appropriate body weight from young adulthood to prevent later impairment of glucose metabolism.

## 2. Materials and Methods

### 2.1. Study Design and Participants

**Figure 1:**
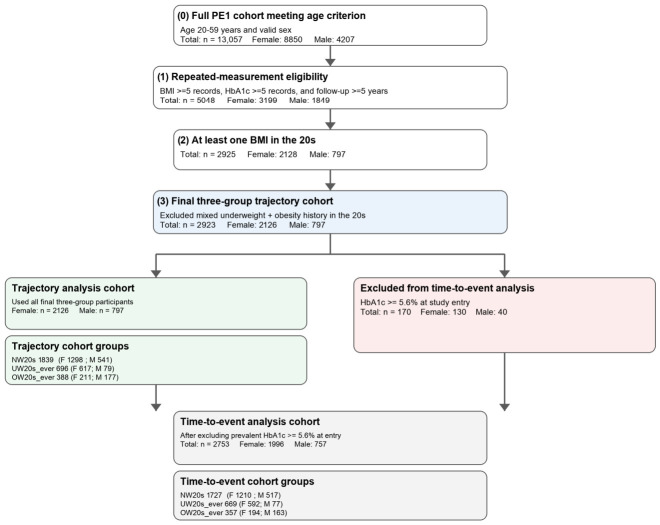
Participant flow for the trajectory and time-to-event analyses. The full cohort in this study included adults aged 20–59 years with valid sex information. Participants were then restricted to those with at least five BMI records, at least five HbA1c records, and a follow-up duration of at least 5 years. Among these, individuals with at least one BMI measurement in their 20s were identified. After exclusion of participants with both underweight and obesity history in their 20s, the final three-group trajectory cohort comprised 2923 participants (2126 women and 797 men), classified as NW20s, UW20s_ever, and OW20s_ever. For the time-to-event analysis, participants with HbA1c ≥ 5.6% at study entry were further excluded, yielding a final event-analysis cohort of 2753 participants (1996 women and 757 men).

We conducted a retrospective longitudinal cohort study of employees at Fujita Health University who underwent routine health examinations between April 2003 and March 2025. This study included adults aged 20–59 years with at least five health examination records (involving BMI and HbA1c measurements) during the observation period (follow-up duration ≥5 years). From the full cohort (*n* = 13,057 individuals, including 8850 women and 4207 men), we identified 2923 individuals (including 2126 women and 797 men) (Figure 1, Table 1). Participants who had both underweight and overweight BMI records during their twenties were excluded, because this mixed-history pattern did not fit the prespecified three-group phenotype framework and was too rare for separate meaningful analysis. The physical data and food frequency questionnaires were provided by the healthcare centre at our university in a fully anonymised form; therefore, the data were depersonalized (accessed on 6 December 2025). In this study, we analyzed data that had already been anonymised, thus making it impossible to identify individuals. For this reason, we have announced on our website of the Department of Clinical Nutrition, Fujita Medical School (approval date: from 21 April 2025 to 31 March 2029) that exclusion is not possible. The study was conducted in accordance with the principles of the Declaration of Helsinki and approved by the Research Ethics Committee of Fujita Health University (application numbers HM25-356: 25 November 2025; HM25-606: 9 March 2026; and HM25-683: 8 April 2026).

### 2.2. Data Collection and Definitions

Health examination data included anthropometric measurements (height and weight) and demographic information (age and sex). BMI was calculated as weight in kilograms divided by height in metres squared (kg/m^2^). BMI was classified into three categories according to the World Health Organization criteria: underweight (BMI < 18.5 kg/m^2^), normal weight (18.5 ≤ BMI < 25.0 kg/m^2^), and obese (BMI ≥ 25.0 kg/m^2^). Age groups were stratified into four 10-year categories: 20s (20–29 years), 30s (30–39 years), 40s (40–49 years), and 50s (50–59 years).

The analysis subjects and group classification analysis subjects were individuals aged 20 to 59 years who had at least five recorded measurements of both BMI and HbA1c, including at least one measurement conducted at the age of 20 years. For the longitudinal analysis of HbA1c (repeated measures analysis), only those measurements with a recording period of five years or more were included. On the basis of their 20-year BMI history, the subjects were classified into three groups: NW20s, UW20s_ever, and OW20s_ever (the baseline characteristics are shown in Table 1). Because underweight status in young adults often fluctuates around the diagnostic cutoff rather than remaining persistently below it, exposure was defined on the basis of BMI history across the twenties rather than a single examination value. Thus, participants were classified as UW20s_ever or OW20s_ever if they met the corresponding BMI criterion at least once during their twenties [18]. The exposure of interest, bodyweight history in the twenties, was conceptualized as a history-based phenotype reflecting cumulative influences of young-adult lifestyle, nutritional status, and metabolic background, rather than as a single anthropometric value alone. Prior to 2013, HbA1c values subtracted by a value of 0.4 were used (HbA1c(JDS)), and values from 2003 to 2013 were adjusted by adding 0.4 to match the HbA1c(NGSP) value.

### 2.3. Longitudinal Analysis of HbA1c Trajectories

#### Longitudinal Analysis of HbA1c Trajectory (Repeated Measures): Estimated Marginal Means (EMMs) and Local Slope

Longitudinal changes in HbA1c were analyzed using a linear mixed-effects model stratified by sex to account for within-individual correlations in repeated measures data. This modelling approach was chosen to account for within-person correlation in repeated HbA1c measurements, between-person heterogeneity, and potential nonlinearity in age-related change, and to derive age-specific model-based estimates such as EMMs and local slopes. The main models were designed to characterize longitudinal HbA1c patterns according to BMI history in the twenties under a sex-stratified framework and were therefore not intended as fully adjusted causal models. Age was treated as a continuous variable, and the age effect was modelled using spline functions (smoothing terms) to allow for nonlinearity, with interaction terms included between age and BMI history groups (NW20s, UW20s_ever, and OW20s_ever). On the basis of the model estimates, the estimated marginal means (EMMs) of HbA1c and their 95% confidence intervals were calculated at the ages of 25, 35, and 45 years. The differences between groups (UW–NW, OW–NW, and OW–UW) at the same age points were evaluated as differences in the EMMs. Using the same model, the local slope (dHbA1c/dAge, % per year) and its 95% confidence interval at the ages of 25, 35, and 45 years were estimated as marginal trends.

In the sensitivity analysis, the hemoglobin level was additionally included in the models because anemia may influence the HbA1c measurements. All analyses were performed using R version 4.5.2 (R Foundation for Statistical Computing, Vienna, Austria).

### 2.4. Time-to-Event Analysis

#### 2.4.1. Time-to-Event Analysis for Initial Attainment of HbA1c ≥ 5.6% (KM/Cox)

HbA1c ≥ 5.6% was used to define dysglycemia on the basis of the threshold used in Japanese health check-up programmes. The outcome was defined as the first subsequent occurrence of HbA1c ≥ 5.6% during follow-up. Study entry was defined as the first examination at which HbA1c was available, and individuals with HbA1c ≥ 5.6% at study entry were excluded. Age was used as the time scale, with entry age defined as the age at the first available HbA1c measurement and exit age defined as the age at the first HbA1c ≥ 5.6% event or, for censored participants, the age at the last available HbA1c measurement. Threshold-based analyses were performed as complementary to trajectory-based findings. The cumulative incidence was estimated using the Kaplan–Meier method, and curves were created for each BMI trajectory group stratified by sex, with the number at risk at each age point also shown. For comparisons between groups, Cox proportional hazards models were fitted with NW20s as the reference group, and hazard ratios (HRs) and 95% confidence intervals (CIs) were estimated for UW20s_ever and OW20s_ever. To assess sex differences, a model including sex-by-group interaction terms was additionally fitted, and an overall interaction test was performed using the likelihood ratio test (LRT). The main estimated results are presented as sex-stratified HRs in a forest plot.

#### 2.4.2. Supplemental Analysis Using Age-Dependent (Time-Varying) HRs

To assess possible age-dependent variation in relative hazards, we additionally fitted sex-stratified time-varying Cox models in which the effects of underweight history and obesity history were allowed to vary smoothly with age through natural spline interactions. Using these models, age-specific HRs and 95% CIs were estimated and plotted against age. Because these estimates represent local effects at each age, they were interpreted separately from the overall Cox HRs estimated across the full follow-up period. In particular, wider confidence intervals at older ages were interpreted cautiously because of the smaller risk set.

#### 2.4.3. Differences Between Trajectory Analysis (EMM/Local Slope) and Time-Varying HR

Trajectory analysis (EMM/local slope) and time-varying HR analysis differ methodologically in terms of their target outcomes and modelling frameworks. In trajectory analysis, repeated measures of HbA1c (continuous outcomes) are used to estimate the trajectory of HbA1c over age via a linear mixed-effects model using calculations of the estimated marginal means (EMMs) at ages of 25, 35, and 45 years, as well as the local slope (dHbA1c/dAge) as the derivative of this curve. In contrast, time-varying HR analysis uses a Cox model with “the first occurrence of HbA1c ≥ 5.6% (time-to-event)” as the outcome and estimates and visualizes the hazard ratio (HR(t)) as a function of age to assess possible changes in effect with age (i.e., deviations from the proportional hazards assumption). This is a different estimator from the “overall HR” shown in the forest plot (which is the average summary across the entire follow-up period). Therefore, it should be noted that, in time-varying HRs, estimates at older ages are more likely to exhibit wider confidence intervals because of the shrinking risk set, and differences arise in how uncertainty is generated, as well as in the interpretive focus compared with trajectory analysis (which estimates the transition of the continuous variables).

The level of significance and estimates were (in principle) presented as point estimates with 95% confidence intervals, and *p* values were evaluated using two-sided tests. EMMs and local slopes were presented as estimates at the ages of 25, 35, and 45 years, and time-to-event analyses were reported along with KM curves and Cox HRs (including stratified and interaction tests).

### 2.5. Ethics

This study was conducted in accordance with the Declaration of Helsinki and was approved by the Research Ethics Committee of Fujita Health University (HM25-356: 25 November 2025; HM25-606: 9 March 2026; and HM25-683: 8 April 2026). The health examination data and food frequency questionnaire data were provided in fully anonymised form. Because the study used anonymised retrospective data, the requirement for informed consent was waived, and an opt-out procedure was implemented through the website of the Department of Clinical Nutrition, Fujita Health University.

## 3. Results

### 3.1. Baseline Characteristics

The analysis included individuals aged 20–59 years who had at least five recorded measurements of both BMI and HbA1c and at least one measurement obtained at the age of 20 years. Furthermore, for the longitudinal analysis of HbA1c trends, only those with a recording period of five years or more were included. After the selection criteria were applied, 2923 participants were ultimately included (2126 women and 797 men). Participants were classified into three groups on the basis of their BMI history in their twenties: NW20s, UW20s_ever, and OW20s_ever. Owing to the structure of the follow-up period, the number of participants eligible for analysis in each age group decreased as age increased. In all the groups, the duration of follow-up and the number of BMI/HbA1c measurements were approximately 10 years and 10 measurements, respectively (Figure 1, Table 1).

### 3.2. Longitudinal Changes in HbA1c

**Figure 2:**
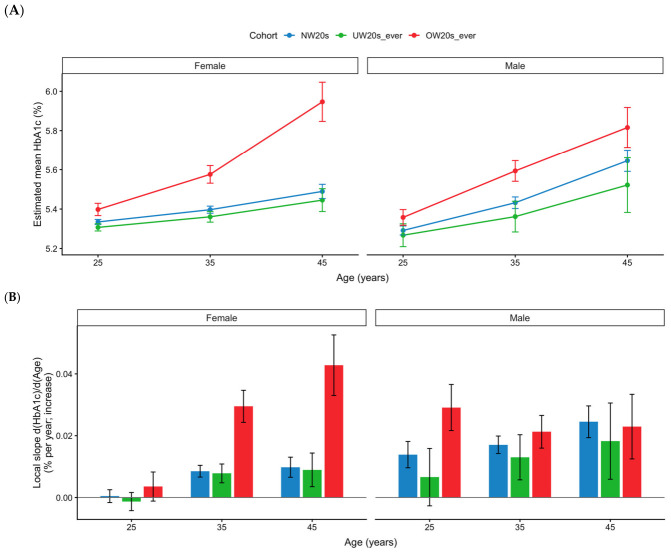
HbA1c trajectories and local slopes at ages 25, 35, and 45 years by BMI-history cohort in the 20s, stratified by sex. (**A**) Estimated mean HbA1c (%) at ages 25, 35, and 45 years with 95% confidence intervals, obtained from a sex-stratified linear mixed-effects model: HbA1c ~ cohort3 × ns (Age) + (1|id). Estimated marginal means (EMMs) of HbA1c with 95% confidence intervals at ages 25, 35, and 45 years were obtained from sex-stratified linear mixed-effects models. Participants with follow-up ≥5 years were included. Cohorts were defined by body-weight history in the 20s: NW20s (no underweight/overweight), UW20s_ever (ever underweight), and OW20s_ever (ever overweight). (**B**) Local slopes (dHbA1c/dAge; % per 1-year increase) at ages 25, 35, and 45 years (95% confidence intervals), derived from marginal trends (emtrends) of the same model. Cohorts were defined using BMI measurements in the 20s: NW20s (no underweight or overweight), UW20s_ever (ever underweight without overweight), and OW20s_ever (ever overweight without underweight). Estimated marginal means and local slopes were computed using the R package (version 4.5.2) emmeans, based on linear mixed-effects models fitted with lme4. OW20s_ever indicates those who experienced being overweight in their 20s. UW20s_ever indicates those individuals who experienced being underweight in their 20s.

The relationship between BMI history in individuals in their 20s and longitudinal changes in HbA1c was assessed using a linear mixed-effects model stratified by sex. Across all groups, HbA1c gradually increased with age, and differences between the groups were observed in the estimated marginal mean (EMM) and local slope (Figure 2, Table 2).

In women, the UW20s_ever group had lower HbA1c EMMs than the NW20s group at ages 25 and 35 years (UW-NW: −0.03 [95% CI: −0.05, −0.01], *p* = 0.015; and −0.04 [−0.07, −0.00], *p* = 0.029, respectively), but not at age 45 years (−0.04 [−0.11, 0.02], *p* = 0.205). In contrast, the OW20s_ever group had higher HbA1c EMMs than the NW20s group at all evaluated ages (OW-NW: 0.06 [0.03, 0.10], *p* < 0.001 at age 25 years; 0.18 [0.13, 0.23], *p* < 0.001 at age 35 years; and 0.46 [0.35, 0.56], *p* < 0.001 at age 45 years). Thus, in women, the difference between OW20s_ever and NW20s increased with age. Consistent with this pattern, the local slopes in women with OW20s_ever were greater at ages 35 and 45 years (0.029 [0.024, 0.035] and 0.043 [0.033, 0.053], respectively) than those in the NW20s and UW20s_ever groups, suggesting a steeper age-related increase in HbA1c.

In men, the OW20s_ever group also had higher HbA1c EMMs than the NW20s group at all evaluated ages (OW-NW: 0.07 [0.02, 0.11], *p* = 0.005 at age 25 years; 0.16 [0.10, 0.22], *p* < 0.001 at age 35 years; and 0.17 [0.05, 0.29], *p* = 0.004 at age 45 years). By contrast, the UW20s_ever group was similar to the NW20s group at all ages (UW-NW: *p* = 0.446, 0.098, and 0.105 at ages 25, 35, and 45 years, respectively). The local slope in men with OW20s_ever was higher at age 25 years (0.029 [0.022, 0.037]) than in the NW20s group (0.014 [0.010, 0.018]), whereas at ages 35 and 45 years the local slopes were broadly similar across groups. These findings indicate that the pattern of age-related HbA1c change in men differed from that in women.

In the sensitivity analysis additionally adjusted for hemoglobin, the estimated HbA1c trajectories were materially unchanged. Across sexes, ages, and BMI-history groups, the differences between the hemoglobin-adjusted and unadjusted EMMs were very small, ranging approximately from −0.006 to +0.004 percentage points, and the overall pattern of between-group differences was preserved (Table 3).

Overall, in women, HbA1c EMMs in the OW20s_ever group were significantly higher than those in the NW20s group at all evaluated ages, and the magnitude of this difference increased with age. The local slopes in women with OW20s_ever were also greater at ages 35 and 45 years, suggesting a steeper age-related increase in HbA1c. In contrast, HbA1c EMMs in the UW20s_ever group were significantly lower than those in the NW20s group at ages 25 and 35 years, but not at age 45 years, and the local slopes were broadly similar to those in the NW20s group. In men, HbA1c EMMs in the OW20s_ever group were higher than those in the NW20s group at all evaluated ages, whereas the local slopes were higher at age 25 years but were broadly similar at ages 35 and 45 years. In the sensitivity analysis additionally adjusted for hemoglobin, the estimated trajectories were materially unchanged, supporting the robustness of the main findings.

### 3.3. Cumulative Incidence of First Occurrence of HbA1c ≥ 5.6%

In the finalized time-to-event analysis, 2753 participants were included after exclusion of 170 participants with HbA1c ≥ 5.6% at study entry. Using age as the time scale, the cumulative incidence of HbA1c ≥ 5.6% and the corresponding hazard ratios were estimated separately for women and men. HbA1c ≥ 5.6% is widely used in Japanese health check-up settings as an early indicator of impaired glucose metabolism and for risk stratification. As a complementary approach to the trajectory-based analysis, we therefore performed threshold-based time-to-event analyses.

**Figure 3:**
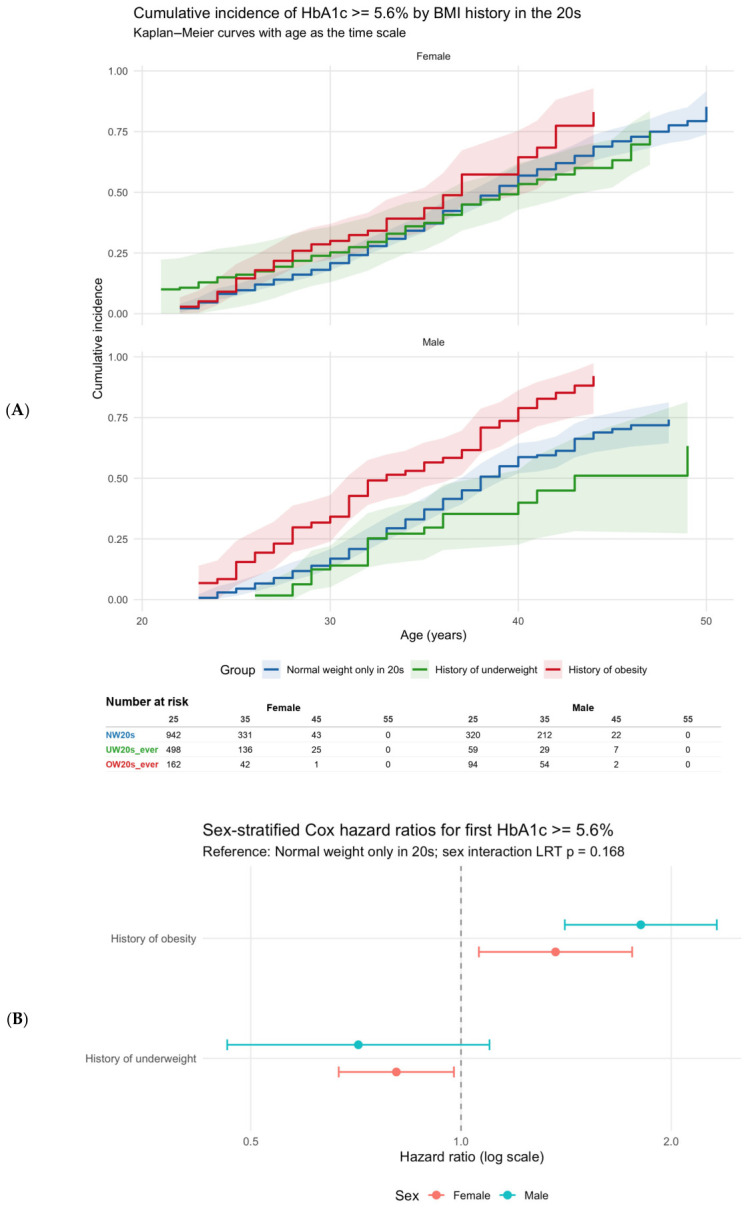

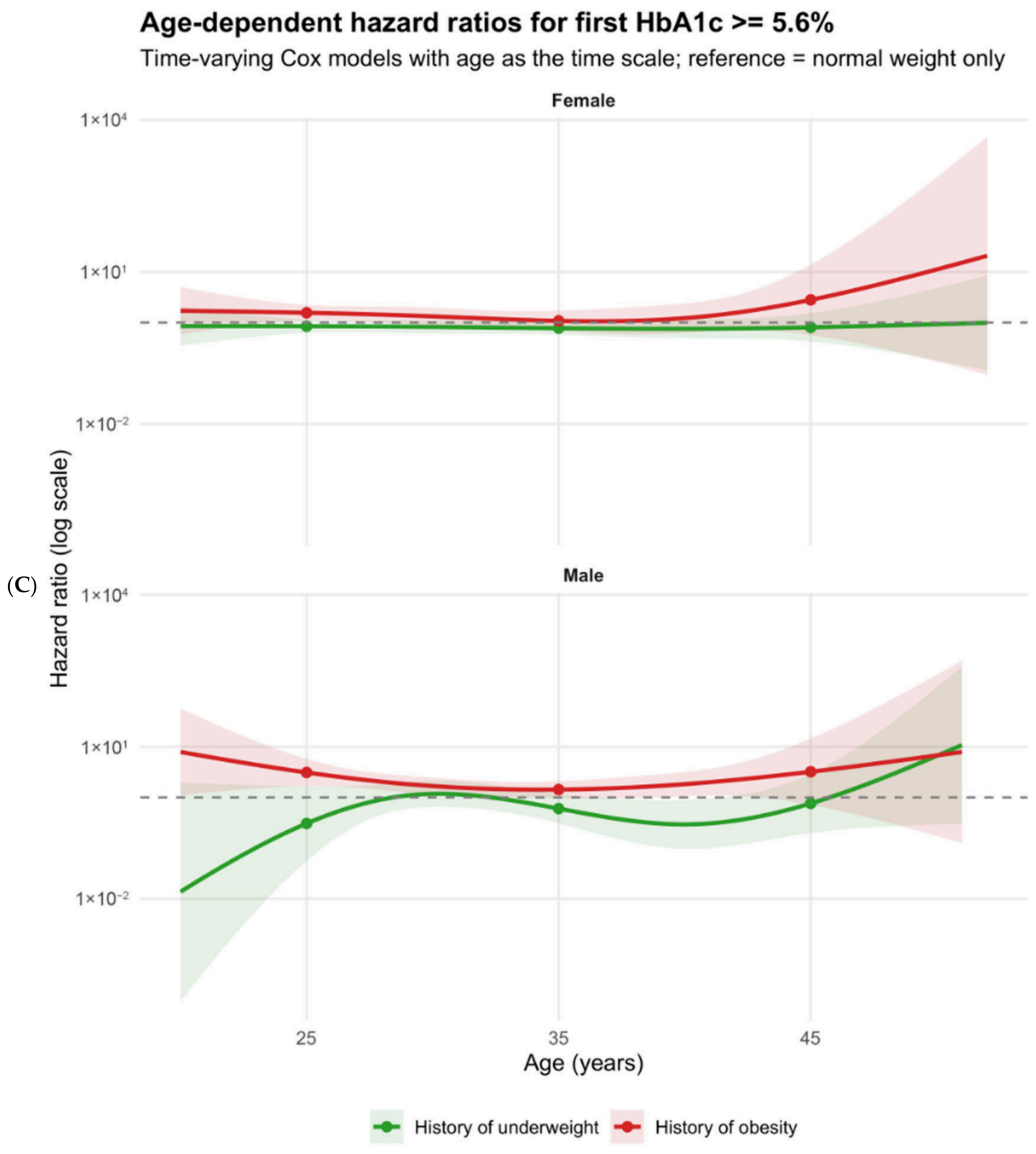
Kaplan–Meier cumulative incidence of incident HbA1c ≥ 5.6% by BMI-history cohort in the 20s, stratified by sex. (**A**) Kaplan–Meier cumulative incidence of reaching HbA1c ≥ 5.6 (supporting analysis). Cumulative incidence (1 − S(t)) is shown using age as the time scale with left truncation (delayed entry at each participant’s first observed age ≥30 years). The event was defined as the first observed HbA1c ≥ 5.6%. Shaded areas indicate 95% confidence intervals. Numbers at risk are displayed below each panel. The inset text reports the log-rank–equivalent *p*-value based on the Cox score test and the sex-specific Cox hazard ratios (HRs) with 95% confidence intervals for UW20s_ever and OW20s_ever compared with NW20s. The numbers at risk indicate the number of participants who remained under observation and had not yet reached HbA1c ≥ 5.6% at each age point. Because the risk set became small at older ages, especially in the obesity history group, estimates in the later age range should be interpreted with caution. (**B**) Forest plot of sex-stratified Cox hazard ratios. Cox proportional hazards regression was performed with age as the time scale using a counting-process formulation to account for left truncation (delayed entry). Participants entered the risk set at their first observed age ≥30 years and were followed until the first observed HbA1c ≥ 5.6% (event) or the last observed HbA1c measurement age (right censoring). Sex-specific models were fit separately for females and males. The dashed vertical line indicates HR = 1. The figure also reports the global interaction test for Sex × Cohort (likelihood ratio test) and interaction *p*-values for UW and OW. Abbreviations: HR, hazard ratio; OW, overweight; UW, underweight. (**C**) Time-varying HR (tt) by age. A secondary model stratified by sex (strata (Sex_group)) was also fit, allowing different baseline hazards by sex while assuming a common cohort effect across sexes. Hazard ratios (HRs) are reported with 95% confidence intervals and two-sided Wald *p*-values. HRs are evaluated at ages 35, 40, 45, and 50 years and plotted on a logarithmic scale (reference HR = 1).

The cumulative incidence of the first occurrence of HbA1c ≥ 5.6% was estimated using the Kaplan–Meier method (Figure 3A). In the sex-stratified Cox analysis, among women, UW20s_ever versus NW20s was associated with a lower risk of reaching HbA1c ≥ 5.6% (HR, 0.81; 95% CI, 0.67–0.98; *p* = 0.028), whereas OW20s_ever versus NW20s was associated with a higher risk (HR, 1.37; 95% CI, 1.06–1.76; *p* = 0.016). Thus, in women, both underweight history and obesity history in the 20s were significantly associated with the risk of attaining HbA1c ≥ 5.6% (Table 4A; Figure 3A,B). In men, UW20s_ever versus NW20s was not significantly associated with the risk of HbA1c ≥ 5.6% (HR, 0.71; 95% CI, 0.46–1.10; *p* = 0.125), whereas OW20s_ever versus NW20s was associated with a significantly higher risk (HR, 1.81; 95% CI, 1.41–2.32; *p* < 0.001). These findings indicate that obesity history in the 20s was associated with a higher risk of incident HbA1c ≥ 5.6% in both women and men, whereas underweight history was associated with a lower risk only in women (Table 4A; Figure 3A,B). To further examine the robustness of the time-to-event findings, we additionally fitted Cox models adjusted for baseline HbA1c. In an additional Cox model adjusted for baseline HbA1c, the association for OW20s_ever remained materially unchanged in both women and men (women: HR 1.42, 95% CI 1.10–1.82; men: HR 1.83, 95% CI 1.42–2.35). By contrast, the inverse association for UW20s_ever in women was attenuated and no longer statistically significant (HR 0.87, 95% CI 0.72–1.05), while the estimate in men remained non-significant (HR 0.70, 95% CI 0.45–1.08). Thus, the association between overweight history in the twenties and later dysglycemia remained robust, whereas the association for underweight history in women was weakened after additional adjustment for baseline HbA1c.

In the interaction model, neither the sex × UW interaction nor the sex × OW interaction was statistically significant (*p* = 0.513 and *p* = 0.106, respectively), and the overall likelihood ratio test for interaction was also not significant (*p* = 0.168). Thus, no statistically significant sex-by-group interaction was observed (Table 4B).

In the age-dependent analysis, the hazard ratios showed some variation over age and became less precise at older ages. For example, in women with an obesity history, the age-specific hazard ratio was 1.56 at age 25 years, 1.08 at age 35 years, and 2.80 at age 45 years. In men with an obesity history, the corresponding values were 3.10, 1.43, and 3.21 (Figure 3C). These age-specific estimates should be interpreted as local effects and therefore do not necessarily coincide with the overall Cox hazard ratios. The confidence intervals widened substantially at older ages, indicating increasing instability of the age-specific estimates in the later age range. Accordingly, caution is warranted when interpreting cohort comparisons at specific older ages, particularly from around age 45 years onward. This pattern was consistent with the marked decrease in the number at risk with advancing age in the Kaplan–Meier analysis (Figure 3A).

Overall, OW was associated with a significantly higher risk of attaining HbA1c ≥ 5.6% in both women and men, whereas UW was associated with a significantly lower risk only in women. Together with the trajectory analysis, these findings suggest that a history of obesity in the 20s is associated with both higher HbA1c levels over time and a greater risk of dysglycemia, particularly in women.

## 4. Discussion

In this retrospective longitudinal study, motivated by previous reports suggesting a possible association between underweight status in young women and the risk of future abnormal glucose metabolism, we examined whether BMI history in young adulthood, specifically in the 20s, was associated with subsequent HbA1c trajectories and the risk of first reaching HbA1c ≥ 5.6%. Our findings suggest sex-related differences in the association between BMI history in the 20s and later HbA1c-related outcomes. Among women, the OW20s_ever group showed consistently higher HbA1c estimated marginal means (EMMs) than the NW20s group at all evaluated ages, and the difference increased with age. In addition, the local slope was greater in the OW20s_ever group, particularly at ages 35 and 45 years, suggesting a steeper age-related increase in HbA1c. By contrast, the UW20s_ever group showed lower HbA1c EMMs than the NW20s group at ages 25 and 35 years, whereas the local slopes were broadly similar between these groups. In the time-to-event analysis, women in the OW20s_ever group had a significantly higher hazard of first reaching HbA1c ≥ 5.6%, whereas women in the UW20s_ever group had a significantly lower hazard, consistent with the trajectory-based findings. Among men, the OW20s_ever group also showed higher HbA1c EMMs than the NW20s group at all evaluated ages, whereas the UW20s_ever group was broadly similar to the NW20s group. However, unlike in women, the local slopes in men were broadly similar across groups at ages 35 and 45 years. In the time-to-event analysis, men in the OW20s_ever group had a significantly higher hazard of first reaching HbA1c ≥ 5.6%, indicating that an overweight history in the 20s was associated with an increased future risk of dysglycemia. In contrast, a history was associated with a lower HbA1c-related risk only in women. Taken together, these findings suggest that an overweight history in the 20s is associated with an increased future risk of HbA1c elevation in both women and men, whereas an underweight history may be associated with a lower HbA1c-related risk only in women. The novelty of the present study lies in demonstrating, through both trajectory-based and time-to-event analyses, that the influences of weight-gain- and weight-loss-related body size history in young adulthood on later HbA1c are sex-dependent and not simply mirror images of one another. In particular, underweight history showed a female-specific association with subsequent HbA1c regulation, whereas overweight history was associated with higher HbA1c-related risk in both sexes.

First, we performed trajectory analysis of HbA1c using linear mixed-effects models. A particular advantage of this approach is that it allows separation of differences in HbA1c level from differences in the rate of age-related HbA1c increase, which could not be captured by simpler longitudinal summaries or threshold-based analyses alone. Regarding the HbA1c trajectory in women, the UW20s_ever group generally showed lower EMMs than the NW20s group at ages 25 and 35 years, whereas the local slopes were broadly similar between the two groups. Consistently, in the time-to-event analysis, the Cox hazard ratio for first reaching HbA1c ≥ 5.6% was significantly lower in women with UW20s_ever. At first glance, these findings may appear inconsistent with our previous cross-sectional study, in which OW was associated with higher HbA1c values in the 20s, 30s, and 40s, whereas UW was broadly similar to NW [30]. However, our more recent work suggested that many women classified as underweight had BMI values close to the lower boundary of the normal range, often around 18.0 kg/m^2^, and frequently moved back and forth between underweight and normal weight across examination years [18]. Thus, in a cross-sectional design, a substantial proportion of women may be classified as underweight in one year and normal weight in another [18]. In this context, defining the exposure longitudinally as a history of underweight in the 20s may better capture the relevant body size phenotype than a single cross-sectional classification.

Previous studies have suggested that underweight young Japanese women may be more likely to have impaired glucose tolerance [29,31]. For example, Tamura et al. reported a higher prevalence of IGT in underweight young Japanese women than in those with standard weight based on a 75 g OGTT [29]. However, this interpretation warrants caution. OGTT and HbA1c do not assess the same dimension of glucose metabolism: OGTT primarily captures the acute handling of an imposed glucose load, whereas HbA1c reflects average glycaemia over time. Therefore, an abnormal OGTT result does not necessarily indicate sustained deterioration of everyday glycaemic status. This distinction may be particularly important in lean populations, in whom a fixed 75 g glucose load may represent a relatively greater physiological challenge. Another report from the same research group suggested that underweight young women do not necessarily differ from women of standard weight in glucose tolerance at younger ages, whereas postmenopausal underweight women may experience a greater decline in glucose tolerance, potentially related to reduced lean mass, increased intramyocellular lipid, and impaired insulin secretion [31]. Therefore, our finding that UW20s_ever was not associated with an adverse longitudinal HbA1c pattern is not necessarily inconsistent with the previous literature when heterogeneity within the underweight group and differences in outcome measures are taken into account. From this perspective, the present longitudinal findings do not support a straightforward interpretation that an underweight history in young women necessarily confers a higher long-term HbA1c-related risk; rather, they suggest that such an interpretation may overstate the long-term glycaemic implications of underweight status when assessed using HbA1c-based outcomes.

A further possible explanation is the influence of female hormonal status [37,38,39,40,41]. Estrogen is known to support insulin action and suppress visceral fat accumulation, making it biologically plausible that the HbA1c profile of women with an underweight history may be relatively favourable before menopause. In the present study, HbA1c EMMs in UW20s_ever were significantly lower than those in NW20s at ages 25 and 35 years, when ovarian hormonal effects are likely to be stronger, but this difference was no longer evident at age 45 years. By contrast, no clear difference between UW20s_ever and NW20s was observed in men at any age. Taken together, these findings suggest that an underweight history in the 20s may be associated with a lower HbA1c-related risk only in women, particularly before midlife, although this interpretation remains hypothesis-generating. Because most women in the present cohort were in the premenopausal age range, longer-term follow-up, including the menopausal transition, will be necessary to clarify whether this pattern changes later in life.

In both women and men, the OW20s_ever group showed higher HbA1c EMMs than the NW20s group, and the Cox hazard ratio for first reaching HbA1c ≥ 5.6% was also higher in the OW20s_ever group. However, the age-related pattern of HbA1c increase differed by sex. In women, the local slope was greater in the OW20s_ever group at ages 35 and 45 years, whereas in men, the corresponding difference was more evident at age 25 years and was less apparent at later ages. These findings suggest that although an overweight history in the 20s was associated with higher future HbA1c-related risk in both sexes, the temporal pattern of HbA1c worsening may differ between women and men.

Several mechanisms may help explain this sex difference, including differences in fat distribution (visceral versus subcutaneous fat), adipose tissue function (such as storage capacity, inflammation, and suppression of lipolysis), and the sex hormone milieu, particularly estrogen [37,38,39,40,41]. These factors suggest that even at a similar BMI, or with a similar history of obesity, the degree of insulin resistance and the metabolic burden on hepatic glucose regulation may differ by sex. In women, the greater capacity for subcutaneous fat storage may initially buffer excess energy and suppress ectopic fat deposition in the liver and skeletal muscle [38,39], thereby attenuating the early metabolic consequences of overweight history. In this context, elevated HbA1c levels and steeper local slopes may become apparent before overt threshold attainment, whereas the increase in hazard for first reaching HbA1c ≥ 5.6% may become more pronounced later. This interpretation may be relevant to our finding that, in women, the local slopes in the OW20s_ever group were greater at ages 35 and 45 years, whereas the age-dependent hazard ratio also appeared to rise again at older ages. Because estrogenic effects decline during the menopausal transition, accompanied by changes in insulin sensitivity and fat distribution [37,40,41], it is plausible that the metabolic consequences of earlier overweight history become more evident around midlife. By contrast, men may be more likely to accumulate visceral and ectopic fat at an earlier stage, leading to earlier metabolic consequences. Because the present study did not include direct measures of body composition, visceral fat, subcutaneous fat, muscle mass, or reproductive hormonal status, future studies incorporating these factors, including menstrual or menopausal status, will be important for a more mechanistic interpretation of the observed sex-specific patterns.

This study has several limitations. First, it was a single-centre retrospective longitudinal study, and selection bias related to healthcare-seeking behaviour, occupational background, and regional characteristics cannot be ruled out. Therefore, the external generalisability of the findings may be limited. On the basis of the present findings, we plan to conduct future replication analyses using larger-scale health check-up datasets.

Second, although the average observation period was approximately 10 years, the risk set decreased with age as follow-up progressed. As a result, estimation precision and statistical power were reduced in older age groups, particularly after approximately 45 years of age, leading to greater uncertainty in age-specific between-group comparisons. Consistent with this, the confidence intervals in the time-varying hazard ratio analysis widened substantially at older ages. Likewise, the EMMs and local slopes presented in the trajectory analysis (Table 2A,B; Figure 2) were model-based estimates derived from repeated-measures longitudinal models, and therefore, estimates in age ranges with relatively sparse observations were more dependent on model specification. Accordingly, estimates in older age groups should be interpreted cautiously, and the overall conclusions should be based primarily on age ranges with higher data density, complemented by both continuous HbA1c trajectories and threshold-based event analyses. Thus, the later-age portions of the time-varying HR curves should be interpreted primarily as exploratory visualizations of possible age-related variation, rather than as definitive age-specific effect estimates. Because detailed and harmonized longitudinal information on several potential confounders was not consistently available, residual confounding cannot be excluded, too.

Third, the event outcome was defined as the first attainment of HbA1c ≥ 5.6%. In Japan, HbA1c ≥ 5.6% is widely used in health check-up settings as an indicator of abnormal glucose metabolism, and thus this threshold has practical relevance in analyses based on routine health examination data. However, from the perspective of disease onset, it would also be important to examine more advanced glycaemic endpoints, such as HbA1c ≥ 6.5%. In the present cohort, the number of such events and the available duration of follow-up were insufficient for stable analysis. In addition, the present exposure definition, particularly for UW20s_ever, was designed to capture underweight history across the twenties rather than to distinguish persistent underweight from fluctuating or nonpersistent underweight. Future studies using larger-scale, multi-centre health check-up databases, including individuals in their 50s and 60s, will be needed to determine whether the present findings extend to more clinically advanced glycaemic outcomes and to evaluate whether persistent and nonpersistent underweight histories differ in their associations with later HbA1c trajectories and threshold attainment.

Moreover, a potential limitation of this study is the possibility of residual confounding by socioeconomic and lifestyle factors. However, body size history in the twenties may itself reflect a complex life-course phenotype comprising lifestyle, behavioural, and metabolic characteristics established in early adulthood. In addition, contemporaneous BMI and baseline HbA1c may partly reflect the long-term metabolic consequences of body size history established in the twenties. Therefore, lifestyle factors, contemporaneous BMI, and baseline HbA1c may act not only as potential confounders but also as downstream mediators or integral components of the life-course exposure of interest. Because the primary aim of this study was to evaluate the overall long-term association of underweight and overweight history in the twenties with subsequent HbA1c regulation, rather than to isolate the independent effects of specific lifestyle or metabolic factors, these variables were not included in the primary models, as adjustment could attenuate or distort the life-course association of interest. In addition, the hemoglobin-adjusted trajectory sensitivity analysis, already shown in Table 3, did not materially alter the main findings, and additional baseline HbA1c-adjusted Cox models likewise supported the robustness of the OW20s_ever association, while suggesting attenuation of the inverse UW20s_ever association in women. In interpreting these findings, it is important to recognize that bodyweight history in the twenties may represent a composite phenotype encompassing not only body size itself but also cumulative lifestyle- and metabolism-related influences during young adulthood.

Accordingly, the findings should be interpreted as longitudinal associations rather than causal effects.

## 5. Conclusions

This study retrospectively examined whether BMI history in the twenties (NW20s/UW20s_ever/OW20s_ever) was associated with subsequent longitudinal changes in HbA1c, assessed by estimated marginal means (EMMs) and local slopes, and with the risk of first reaching HbA1c ≥ 5.6%. In women, the HbA1c EMMs in UW20s_ever at ages 25 and 35 years were significantly lower than those in NW20s, and UW20s_ever was also associated with a lower Cox hazard for HbA1c ≥ 5.6%. By contrast, OW20s_ever was associated with higher HbA1c levels in both women and men. However, the age-related pattern differed by sex: in men, the local slope in OW20s_ever was greater than that in NW20s mainly at age 25 years, whereas in women, the local slope in OW20s_ever remained greater at older ages. These findings suggest that underweight and overweight histories in the twenties are not simply mirror-image exposures, but rather they have sex-dependent and asymmetric associations with later HbA1c regulation. In particular, overweight history in the twenties was associated with a higher future HbA1c-related risk in both sexes, whereas underweight history was associated with a lower HbA1c-related risk only in women. This represents a novel longitudinal perspective that could not be captured by cross-sectional analyses alone. Future large-scale studies incorporating body composition, fat distribution, and menstrual or other reproductive hormonal information will be needed to clarify the mechanisms underlying these sex- and age-related differences.

## Figures and Tables

**Table 1:** Baseline data of the participants in this study.

Variable	Trajectory Cohort				Time-to-Event Cohort			
Variable	NW20s	UW20s_Ever	OW20s_Ever	*p*	NW20s	UW20s_Ever	OW20s_Ever	*p*
Participants, *n*	F: 1298 M: 541	F: 617 M: 79	F: 211 M: 177		F: 1210 M: 517	F: 592 M: 77	F: 194 M: 163	
Age at baseline, mean (SD), years	F: 23.23 (2.30) M: 24.75 (2.45)	F: 22.89 (1.96) M: 23.90 (2.36)	F: 22.67 (2.11) M: 24.67 (2.44)	F: <0.001 M: 0.008	F: 23.24 (2.31) M: 24.74 (2.45)	F: 22.89 (1.96) M: 23.94 (2.37)	F: 22.62 (2.08) M: 24.71 (2.45)	F: <0.001 M: 0.013
BMI at baseline, mean (SD), kg/m^2^	F: 20.93 (1.41) M: 21.56 (1.59)	F: 18.47 (1.14) M: 18.32 (1.01)	F: 26.04 (2.91) M: 26.43 (3.52)	F: <0.001 M: <0.001	F: 20.93 (1.41) M: 21.57 (1.59)	F: 18.47 (1.13) M: 18.34 (1.02)	F: 25.96 (2.88) M: 26.42 (3.48)	F: <0.001 M: <0.001
HbA1c at baseline, mean (SD), %	F: 5.34 (0.24) M: 5.28 (0.24)	F: 5.31 (0.21) M: 5.26 (0.26)	F: 5.39 (0.48) M: 5.32 (0.26)	F: 0.005 M: 0.039	F: 5.31 (0.21) M: 5.26 (0.21)	F: 5.30 (0.20) M: 5.25 (0.25)	F: 5.32 (0.22) M: 5.29 (0.25)	F: 0.062 M: 0.163
Hemoglobin at baseline, mean (SD), g/dL	F: 12.82 (1.00) M: 15.21 (0.84)	F: 12.88 (1.04) M: 14.99 (1.43)	F: 13.17 (0.92) M: 15.45 (0.92)	F: <0.001 M: <0.001	F: 12.85 (0.98) M: 15.21 (0.84)	F: 12.91 (1.01) M: 14.99 (1.45)	F: 13.16 (0.89) M: 15.47 (0.92)	F: <0.001 M: <0.001
Follow-up, median [Q1, Q3], years	F: 8.00 [6.00, 14.00] M: 10.00 [7.00, 14.00]	F: 8.00 [6.00, 12.00] M: 10.00 [7.00, 15.00]	F: 9.00 [6.00, 12.00] M: 9.00 [7.00, 12.00]	F: 0.140 M: 0.272	F: 8.00 [6.00, 14.00] M: 10.00 [7.00, 15.00]	F: 8.00 [6.00, 13.00] M: 10.00 [7.00, 15.00]	F: 9.00 [6.00, 13.00] M: 9.00 [7.00, 12.00]	F: 0.140 M: 0.326
Total visits, median [Q1, Q3]	F: 10.00 [8.00, 15.00] M: 11.00 [8.00, 16.00]	F: 9.00 [7.00, 14.00] M: 12.00 [8.00, 18.00]	F: 10.00 [8.00, 15.00] M: 10.00 [8.00, 13.00]	F: 0.085 M: 0.049	F: 10.00 [8.00, 15.00] M: 11.00 [8.00, 16.00]	F: 9.00 [7.00, 14.00] M: 12.00 [9.00, 18.00]	F: 10.00 [8.00, 15.00] M: 10.00 [8.00, 13.00]	F: 0.126 M: 0.049
BMI visits, median [Q1, Q3]	F: 9.00 [7.00, 14.00] M: 10.00 [8.00, 15.00]	F: 9.00 [7.00, 13.00] M: 12.00 [8.00, 17.00]	F: 9.00 [7.00, 14.00] M: 10.00 [8.00, 13.00]	F: 0.119 M: 0.066	F: 9.00 [7.00, 15.00] M: 11.00 [8.00, 16.00]	F: 9.00 [7.00, 13.00] M: 12.00 [8.00, 17.00]	F: 9.00 [7.00, 15.00] M: 10.00 [8.00, 13.00]	F: 0.186 M: 0.064
HbA1c visits, median [Q1, Q3]	F: 9.00 [7.00, 13.00] M: 10.00 [8.00, 15.00]	F: 9.00 [7.00, 13.00] M: 11.00 [8.00, 17.00]	F: 9.00 [7.50, 13.50] M: 9.00 [7.00, 12.00]	F: 0.071 M: 0.057	F: 9.00 [7.00, 14.00] M: 10.00 [8.00, 15.00]	F: 9.00 [7.00, 13.00] M: 11.00 [8.00, 17.00]	F: 9.00 [7.00, 14.00] M: 10.00 [7.00, 12.00]	F: 0.119 M: 0.066
Time to event/censor, median [Q1, Q3], years	F: 7.00 [5.00, 11.00] M: 8.00 [6.00, 11.00]	F: 7.00 [5.00, 11.00] M: 8.00 [6.00, 13.00]	F: 7.00 [5.00, 11.00] M: 7.00 [4.00, 10.00]	F: 0.333 M: <0.001	F: 7.00 [5.00, 11.00] M: 8.00 [6.00, 12.00]	F: 7.00 [5.00, 11.00] M: 9.00 [6.00, 13.00]	F: 7.00 [5.00, 11.00] M: 7.00 [5.00, 11.00]	F: 0.575 M: <0.001
Incident HbA1c ≥ 5.6%, *n* (%)	F: 479 (36.9%) M: 224 (41.4%)	F: 172 (27.9%) M: 25 (31.6%)	F: 89 (42.2%) M: 103 (58.2%)	F: <0.001 M: <0.001	F: 391 (32.3%) M: 200 (38.7%)	F: 147 (24.8%) M: 23 (29.9%)	F: 72 (37.1%) M: 89 (54.6%)	F: <0.001 M: <0.001

Data are represented as mean (SD). Abbreviations: NW20s, People in their 20s who have always been of normal weight; UW20s_ever, those who have ever been underweight even once in their 20s; OW20s_ever, those who have ever been obese even once in their 20s; BMI, body mass index.

**Table 2:** Estimated marginal means (EMMs) and local slopes of HbA1c by BMI-history cohort in the 20s, stratified by sex. (**A**) EMMs of HbA1c at ages 25, 35, and 45 years with 95% confidence intervals. (**B**) Local slopes (dHbA1c/dAge) at ages 25, 35, and 45 years with 95% confidence intervals, derived from the same spline-based mixed-effects model.

(A) Estimated Marginal Means (EMMs) of HbA1c and Pairwise Differences by Cohort, Strati-Fied by Sex								
Sex	Age	*n*	NW20s	UW20s_Ever	OW20s_Ever	UW–NW	OW–NW	OW–UW
Female	25	1800	5.33 (5.32, 5.35)	5.31 (5.29, 5.33)	5.40 (5.37, 5.43)	**−0.03 (−0.05, −0.01); *p* = 0.015**	**0.06 (0.03, 0.10); *p* < 0.001**	**0.09 (0.05, 0.13); *p* < 0.001**
Female	35	686	5.40 (5.38, 5.41)	5.36 (5.33, 5.39)	5.58 (5.53, 5.62)	**−0.04 (−0.07, −0.00); *p* = 0.029**	**0.18 (0.13, 0.23); *p* < 0.001**	**0.22 (0.16, 0.27); *p* < 0.001**
Female	45	144	5.49 (5.45, 5.53)	5.45 (5.39, 5.50)	5.95 (5.85, 6.05)	−0.04 (−0.11, 0.02); *p* = 0.205	**0.46 (0.35, 0.56); *p* < 0.001**	**0.50 (0.39, 0.62); *p* < 0.001**
Male	25	508	5.29 (5.27, 5.31)	5.27 (5.21, 5.33)	5.36 (5.32, 5.40)	−0.02 (−0.09, 0.04); *p* = 0.446	**0.07 (0.02, 0.11); *p* = 0.005**	**0.09 (0.02, 0.16); *p* = 0.012**
Male	35	411	5.43 (5.40, 5.46)	5.36 (5.28, 5.44)	5.59 (5.54, 5.65)	−0.07 (−0.15, 0.01); *p* = 0.098	**0.16 (0.10, 0.22); *p* < 0.001**	**0.23 (0.14, 0.33); *p* < 0.001**
Male	45	75	5.65 (5.59, 5.70)	5.52 (5.38, 5.66)	5.82 (5.71, 5.92)	−0.12 (−0.27, 0.03); *p* = 0.105	**0.17 (0.05, 0.29); *p* = 0.004**	**0.29 (0.12, 0.47); *p* < 0.001**
**(B) Local Slopes of HbA1c by Cohort, Stratified by Sex**								
**Sex**	**Age**	***n***	**NW20s**		**UW20s_Ever**		**OW20s_Ever**	
Female	25	1800	0.000 (−0.002, 0.003)		−0.001 (−0.004, 0.002)		0.004 (−0.001, 0.008)	
Female	35	686	0.009 (0.007, 0.010)		0.008 (0.005, 0.011)		0.029 (0.024, 0.035)	
Female	45	144	0.010 (0.007, 0.013)		0.009 (0.004, 0.014)		0.043 (0.033, 0.053)	
Male	25	508	0.014 (0.010, 0.018)		0.007 (−0.003, 0.016)		0.029 (0.022, 0.037)	
Male	35	411	0.017 (0.014, 0.020)		0.013 (0.006, 0.020)		0.021 (0.016, 0.027)	
Male	45	75	0.025 (0.019, 0.030)		0.018 (0.006, 0.031)		0.023 (0.012, 0.033)	

Footnote: Values are estimated marginal means (EMMs) of HbA1c with 95% confidence intervals (CIs) from sex-stratified linear mixed-effects models fit in the analytic sample (age 20–59 years; follow-up ≥5 years defined as max(Year) − min(Year) ≥ 5; ≥5 non-missing BMI and ≥5 non-missing HbA1c measurements; and ≥1 BMI measurement in the 20s). BMI-history cohorts were defined using BMI in the 20s as: NW20s (no underweight [BMI < 18.5] and no overweight [BMI ≥ 25]), UW20s_ever (ever underweight without overweight), and OW20s_ever (ever overweight without underweight); participants with both underweight and overweight in the 20s were excluded. Models were: HbA1c~cohort3 × ns (Age, *df* = 4) + (1|ID), where ns() denotes a natural cubic spline for age; no additional covariates were included. HbA1c values measured in 2003–2013 were corrected by +0.4. *n*_total indicates the number of unique participants with an observed (non-missing) HbA1c value at the specified age (multiple measurements at the same age within an individual were averaged prior to modelling). Pairwise differences (Diff) are Tukey-adjusted. The 95% CIs for EMMs and contrasts were obtained from emmeans (emmeans::confint), using t-based (Wald-type) intervals with degrees of freedom as implemented for lmer models (Satterthwaite approximation via lmerTest/emmeans). Bold letters indicate significant (*p* < 0.05).

**Table 3:** Estimated means by age and cohort group, shown separately for female and male, from the main model and the model additionally adjusted for hemoglobin.

Sex	Age	Group	Unadjusted EMM (95% CI)	Hb-Adjusted EMM (95% CI)	Delta
Female	25	NW20s	5.33 (5.32, 5.35)	5.34 (5.32, 5.35)	0.001
Female	25	UW20s_ever	5.31 (5.29, 5.33)	5.31 (5.29, 5.33)	0.002
Female	25	OW20s_ever	5.40 (5.37, 5.43)	5.40 (5.37, 5.43)	0.004
Female	35	NW20s	5.40 (5.38, 5.41)	5.39 (5.38, 5.41)	−0.003
Female	35	UW20s_ever	5.36 (5.33, 5.39)	5.36 (5.33, 5.39)	−0.002
Female	35	OW20s_ever	5.58 (5.53, 5.62)	5.57 (5.53, 5.62)	−0.001
Female	45	NW20s	5.49 (5.45, 5.53)	5.49 (5.45, 5.52)	−0.004
Female	45	UW20s_ever	5.45 (5.39, 5.50)	5.44 (5.38, 5.50)	−0.006
Female	45	OW20s_ever	5.95 (5.85, 6.05)	5.94 (5.84, 6.04)	−0.004
Male	25	NW20s	5.29 (5.27, 5.31)	5.29 (5.27, 5.31)	0.001
Male	25	UW20s_ever	5.27 (5.21, 5.33)	5.27 (5.21, 5.32)	−0.000
Male	25	OW20s_ever	5.36 (5.32, 5.40)	5.36 (5.32, 5.40)	0.002
Male	35	NW20s	5.43 (5.40, 5.46)	5.43 (5.40, 5.46)	−0.001
Male	35	UW20s_ever	5.36 (5.28, 5.44)	5.36 (5.28, 5.44)	−0.001
Male	35	OW20s_ever	5.59 (5.54, 5.65)	5.59 (5.54, 5.65)	0.001
Male	45	NW20s	5.65 (5.59, 5.70)	5.64 (5.59, 5.70)	−0.002
Male	45	UW20s_ever	5.52 (5.38, 5.66)	5.52 (5.38, 5.66)	−0.003
Male	45	OW20s_ever	5.82 (5.71, 5.92)	5.81 (5.71, 5.92)	−0.001

Delta indicates the difference between Unadjusted EMM minus Hb-adjusted EMM.

**Table 4:** Time-to-event summary for incident HbA1c ≥ 5.6% according to BMI history in the 20s.

(A) Time-to-Event Summary by Sex and Cohort (Entry56_Event56)						
Sex	Cohort	*n* (Events)	Median Follow-Up Time (IQR)	Event Rate (per 1000 Person-Years)	Cox HR (95% CI)	Cox *p*
Female	NW20s (ref)	1210 (391)	7.00 (5.00–11.00)	36.60	1.00 (ref)	
Female	UW20s_ever	592 (147)	7.00 (5.00–11.00)	28.60	0.81 (0.67–0.98)	0.028
Female	OW20s_ever	194 (72)	7.00 (5.00–11.00)	44.89	1.37 (1.06–1.76)	0.016
Male	NW20s (ref)	517 (200)	8.00 (6.00–12.00)	41.53	1.00 (ref)	
Male	UW20s_ever	77 (23)	9.00 (6.00–13.00)	28.86	0.71 (0.46–1.10)	0.125
Male	OW20s_ever	163 (89)	7.00 (5.00–11.00)	70.52	1.81 (1.41–2.32)	<0.001
**(B) Risk Set and Interaction**						
**Sex**	**Cohort**	**Number at Risk (25/35/45/55 Years)**		**Interaction *p* (Sex × UW)**	**Interaction *p* (Sex × OW)**	
Female	NW20s (ref)	942/331/43/0				
Female	UW20s_ever	498/136/25/0		0.513		
Female	OW20s_ever	162/42/1/0			0.106	
Male	NW20s (ref)	320/212/22/0				
Male	UW20s_ever	59/29/7/0		0.513		
Male	OW20s_ever	94/54/2/0			0.106	

Note. Study entry was defined as the first examination at which HbA1c was available. Participants with HbA1c ≥ 5.6% at study entry were excluded. Age was used as the time scale. Number at risk indicates participants who had entered observation by each age point and remained event-free and uncensored at that age. Interaction *p*-values for Sex × UW and Sex × OW were obtained from the sex-by-group interaction model; the global interaction *p*-value was obtained by the likelihood ratio test.

## Data Availability

The datasets presented in this article are not readily available because the data are part of an ongoing study. Requests to access the datasets should be directed to the corresponding author upon reasonable request.
